# Supplementary material for: A Tutorial on Optimal Dynamic Treatment Regimes
Source: Stat Med. 2026 Feb 4;45(3-5):e70395. doi: 10.1002/sim.70395 (PMC12872042; doi:10.1002/sim.70395)
Supplement: Supplementary file 1 — Data S1: Additional supporting information may be found in the online version in the Supporting Information section at the end of this article. [file SIM-45-0-s001.zip › supplement_R_code.pdf]

Supplementary example code to 'A tutorial on optimal dynamic treatment regimes'

Chunyu Wang and Brian DM Tom

1. Example code for simulation studies in Section 4.1

Load packages

We firstly load the required R packages among which **DTRreg** (https://CRAN.R-project.org/package=DTRreg) works for Q-learning, A-learning and dWOLS, **htetree** (https://CRAN.R-project.org/package=htetree) works for the causal tree method and **DTRlearn2** (https://CRAN.R-project.org/package=DTRlearn2) works for outcome weighting learning (OWL) illustrated in case 2.

```
library(DTRreg) ##package for Q-learning, A-learning and dWOLS.
library(htetree) ##package for causal tree
library(rgenoud) ##package for optimization in IPW and AIPW
library(rpart) ##package for conventional classification and regression trees
library(rpart.plot) ##package for tree plot
library(TruncatedNormal)##package for truncated normal distribution
library(DTRlearn2) ##package for OWL
library(kernlab)
###Define functions
expit=function(x) 1/(1+exp(-x))
```

Generate data

Data is generated as follows.

```
set.seed(1)
n=500
L1=rnorm(n,mean=450,sd=150)
A1=rbinom(n,1,expit(2-0.006*L1))
L2=rnorm(n,mean=1.25*L1,sd=60)
A2=rbinom(n,1,expit(0.8-0.004*L2))
Y=rnorm(n,mean=400+1.6*L1,sd=60)-((L1<=250)-A1)*(250-L1)-((L2<=360)-A2)*(720-2*L2)
data=data.frame(L1=L1,A1=A1,L2=L2,A2=A2,Y=Y)
head(data,n=3) ##The three random individuals shown in Table 1 in the tutorial.
```

```
##          L1 A1       L2 A2         Y
## 1 356.0319  0 357.7584  0 1016.1706
## 2 477.5465  0 546.1939  0 1108.5556
## 3 324.6557  1 330.7909  1  898.4083
```

For illustrative purposes only, we here keep the seed fixed and all statistical methods shown below are applied to the generated dataset under the fixed seed.

Q-learning

To illustrate the backward induction procedure, we firstly show the self-written code for Q-learning followed by directly calling the function 'DTRreg' from package **DTRreg**. The standard error returned from 'DTRreg' for Q-learning is calculated by bootstrap.

```
lm_2_q=lm(Y~A2+A2:L2+L1+A1+A1:L1+L2)
psi_2_q=lm_2_q$coefficients[c(2,6)]
beta_2_q=lm_2_q$coefficients[-c(2,6)]
#design matrix for the blip function in stage 2
blip_mat2=rbind(numeric(n)+1,L2)
#design matrix for the treatment-free term in stage 2
trtfre_mat2_q=rbind(numeric(n)+1,L1,A1,L2,A1*L1)
##construct the value function which serves as the response variable in stage 1
V_2=(c(t(blip_mat2)%*%psi_2_q)>0)*c(t(blip_mat2)%*%psi_2_q)+t(trtfre_mat2_q)%*%beta_2_q
lm_1_q=lm(V_2~A1+A1:L1+L1)
psi_1_q=lm_1_q$coefficients[c(2,4)]
psi_q=c(psi_1_q,psi_2_q)
psi_q
```

```
##           A1       A1:L1          A2       A2:L2
## 167.1323922  -0.8175578 479.8823890  -1.5379876
```

```
#use package
blip.mod=list(~L1,~L2)
treat.mod=list(A1~L1,A2~L2)
tf.mod=list(~L1, ~L1+A1+A1:L1+L2)
res_DTR_q=DTRreg(Y, blip.mod, treat.mod,tf.mod,data=data,
                 method = "qlearn",var.estim = "bootstrap")
summary(res_DTR_q)
```

```
## DTR estimation over  2  stages:
##
##  Blip parameter estimates
## Stage 1 (n = 500)
##       Estimate Std_Error X95._CI_Lower X95._CI_Upper
## A1    167.1324   37.3280       93.9695      240.2952
## L1:A1  -0.8176    0.0837       -0.9816       -0.6535
## Stage 2 (n = 500)
##       Estimate Std_Error X95._CI_Lower X95._CI_Upper
## A2    479.8824   68.7882      345.0575      614.7073
## L2:A2  -1.5380    0.1259       -1.7847       -1.2913
##
## Warning: possible non-regularity at stages 1, 2 (probs = 0.266, 0.288, respectively)
## Recommended dynamic treatment regimen:
## Stage 1: treat if 167.1324  - 0.8176 L1:A1 > 0
## Stage 2: treat if 479.8824  - 1.5380 L2:A2 > 0
```

A-learning

- **A1**: A-learning without the treatment-free term.

Since the function 'DTRreg' does not allow for a null model for the treatment-free term, we wrote the following R code for A1, which is intended to solve the estimating equations:

$$\sum_{i=1}^{n} R_{ji}\left[\tilde{V}_{j+1,i}^{A} - A_{ji}R_{ji}^{\top}\psi_j\right]\left[A_{ji} - \mathbb{E}\left(A_{ji}|H_{ji};\hat{\alpha}_j\right)\right] = 0, \quad j = 1, 2;$$

with $R_{ji} = (1, L_{ji})^{\top}$ for $j = 1, 2$ and $i = 1, \ldots, n$; from which we can get the closed form of $\hat{\psi}_j$ as follows

$$\hat{\psi}_j = \left[\sum_{i=1}^{n} R_{ji}\underbrace{A_{ji}\left[A_{ji} - \mathbb{E}\left(A_{ji}|H_{ji};\hat{\alpha}_j\right)\right]}_{W_j}R_{ji}^{\top}\right]^{-1}\left(\sum_{i=1}^{n} R_{ji}\left[A_{ji} - \mathbb{E}\left(A_{ji}|H_{ji};\hat{\alpha}_j\right)\right]\tilde{V}_{j+1,i}^{A}\right).$$

```
trt_1=glm(A1~L1,family = binomial)
trt_2=glm(A2~L2,family = binomial)
pre_trt_1=predict(trt_1,type = "response")
pre_trt_2=predict(trt_2,type = "response")
##stage 2
W2=(A2-pre_trt_2)*A2
blip_mat2=rbind(numeric(n)+1,L2)
trt_mat2=blip_mat2
est_mat2=tcrossprod(t(t(trt_mat2)*W2),blip_mat2)
est_vec2=rowSums(t(t(trt_mat2)*((A2-pre_trt_2)*Y)))
psi_2=solve(est_mat2,est_vec2)
### stage 1
W1=(A1-pre_trt_1)*A1
blip_mat1=rbind(numeric(n)+1,L1)
trt_mat1=blip_mat1
est_mat1=tcrossprod(t(t(trt_mat1)*W1),blip_mat1)
reg_2=(((psi_2[1]+psi_2[2]*L2)>=0)-A2)*(psi_2[1]+psi_2[2]*L2)
est_vec1=rowSums(t(t(trt_mat1)*((A1-pre_trt_1)*(Y+reg_2))))
psi_1=solve(est_mat1,est_vec1)
psi=c(psi_1,psi_2)
psi
```

```
##                    L1                     L2
## 310.234323  -1.142173 727.184509  -2.005079
```

And the standard error of estimates from A1 can be calculated by the sandwich variance estimator as follows.

```
#standard error of the estimate of psi_2 from A1
R_out2=c(Y-A2*t(blip_mat2)%*%psi_2)
R_trt2=A2-pre_trt_2
U_psi2=matrix(c(R_out2*R_trt2,(R_out2*R_trt2)*L2),nrow=2,ncol=n,byrow = TRUE)
W2_var=R_out2*pre_trt_2*(1-pre_trt_2)
U_psi2_d_alpha2=-tcrossprod(t(t(blip_mat2)*W2_var),trt_mat2)/n
U_alpha2_d_alpha2=-tcrossprod(t(t(trt_mat2)*(pre_trt_2*(1-pre_trt_2))),trt_mat2)/n
U_alpha2=matrix(c(R_trt2,R_trt2*L2),nrow=2,ncol=n,byrow = TRUE)
U_psi2_adj=U_psi2-U_psi2_d_alpha2%*%solve(U_alpha2_d_alpha2)%*%U_alpha2
meat_2=tcrossprod(U_psi2_adj)/n
U_psi2_d_psi2=-tcrossprod(t(t(blip_mat2)*(A2*R_trt2)),trt_mat2)/n
bread_2=solve(U_psi2_d_psi2)
var_psi2=bread_2%*%meat_2%*%t(bread_2)/n
```

```
sd_psi2=sqrt(diag(var_psi2))
sd_psi2
```

```
##                        L2
## 154.8512334   0.3017955
```

```
#standard error of the estimate of psi_1 from A1
R_out1=c(Y+reg_2-A1*t(blip_mat1)%*%psi_1)
R_trt1=A1-pre_trt_1
U_psi1=matrix(c(R_out1*R_trt1,(R_out1*R_trt1)*L1),nrow=2,ncol=n,byrow = TRUE)
U_psi1_d_psi1=-tcrossprod(t(t(blip_mat1)*(A1*R_trt1)),trt_mat1)/n
W1_var=R_out1*pre_trt_1*(1-pre_trt_1)
U_psi1_d_alpha1=-tcrossprod(t(t(blip_mat1)*W1_var),trt_mat1)/n
U_alpha1_d_alpha1=-tcrossprod(t(t(trt_mat1)*(pre_trt_1*(1-pre_trt_1))),trt_mat1)/n
U_alpha1=matrix(c(R_trt1,R_trt1*L1),nrow=2,ncol=n,byrow = TRUE)
U_psi1_d_psi2=tcrossprod(t(t(blip_mat1)*((((psi_2[1]+psi_2[2]*L2)>=0)-A2)*R_trt1)),
                            blip_mat2)/n

U_psi1_adj=U_psi1-U_psi1_d_psi2%*%bread_2%*%U_psi2_adj-U_psi1_d_alpha1%*%
  solve(U_alpha1_d_alpha1)%*%U_alpha1
meat_1=tcrossprod(U_psi1_adj)/n
bread_1=solve(U_psi1_d_psi1)
var_psi1=bread_1%*%meat_1%*%t(bread_1)/n
sd_psi1=sqrt(diag(var_psi1))
sd_psi1
```

```
##                        L1
## 111.6003922   0.2581702
```

- **A2**: regression adjustment for propensity score; that is, the OLS regression of $\tilde{V}_{j+1}^{A}$ on $A_j R_j$ and $\mathbb{E}\left(A_j|H_j,\hat{\alpha}_j\right) R_j$. The estimate of ψ from A2 is given below.

```
lm_2=lm(Y~-1+A2+A2:L2+pre_trt_2+pre_trt_2:L2)
psi_2_lm=lm_2$coefficients[c(1,3)]
reg_2_lm=(((psi_2_lm[1]+psi_2_lm[2]*L2)>=0)-A2)*(psi_2_lm[1]+psi_2_lm[2]*L2)
lm_1=lm((Y+reg_2_lm)~-1+A1+A1:L1+pre_trt_1+pre_trt_1:L1)
psi_1_lm=lm_1$coefficients[c(1,3)]
psi_lm=c(psi_1_lm,psi_2_lm)
psi_lm
```

```
##         A1      A1:L1         A2      A2:L2
## 377.310295  -1.286277 752.108533  -2.063148
```

And the standard errors are calculated as follows.

```
#standard error of the estimate of psi_2 from A2
eta2=lm_2$coefficients[-c(1,3)]
R_out2_lm=c(Y-A2*t(blip_mat2)%*%psi_2_lm-pre_trt_2*t(blip_mat2)%*%eta2)
U_psi2_lm=matrix(c(R_out2_lm*A2,R_out2_lm*(A2*L2)),nrow=2,ncol=n,byrow = TRUE)
U_psi2_d_psi2_lm=-tcrossprod(t(t(blip_mat2)*A2),blip_mat2)/n
U_psi2_d_eta2=-tcrossprod(t(t(blip_mat2)*(A2*pre_trt_2)),blip_mat2)/n
```

```
W2_var_lm=pre_trt_2*(1-pre_trt_2)*A2*c(t(blip_mat2)%*%eta2)
U_psi2_d_alpha2_lm=-tcrossprod(t(t(blip_mat2)*W2_var_lm),trt_mat2)/n

U_eta2=matrix(c(R_out2_lm*pre_trt_2,R_out2_lm*(pre_trt_2*L2)),nrow=2,
              ncol=n,byrow = TRUE)
U_eta2_d_eta2=-tcrossprod(t(t(blip_mat2)*(pre_trt_2^2)),blip_mat2)/n
eta2_mat=tcrossprod(t(t(blip_mat2)*(pre_trt_2^2)),blip_mat2)/n
eta2_vec=t(pre_trt_2*t(blip_mat2))%*%c(Y-A2*t(blip_mat2)%*%psi_2_lm)/n
eta2_d_psi2=-solve(eta2_mat)%*%(tcrossprod(t(t(blip_mat2)*(A2*pre_trt_2)),
                                           blip_mat2)/n)
W_alpha2=pre_trt_2*(1-pre_trt_2)
eta2_d_alpha2_1=-solve(eta2_mat)%*%(tcrossprod(t(t(blip_mat2)*(2*pre_trt_2*W_alpha2)),
                                               blip_mat2)/n)%*%solve(eta2_mat)%*%eta2_vec+
  solve(eta2_mat)%*%t(W_alpha2*t(blip_mat2))%*%c(Y-A2*t(blip_mat2)%*%psi_2_lm)/n

eta2_d_alpha2_2=-solve(eta2_mat)%*%(tcrossprod(t(t(blip_mat2)*(2*pre_trt_2*W_alpha2*L2)),
                                               blip_mat2)/n)%*%solve(eta2_mat)%*%eta2_vec+
  solve(eta2_mat)%*%t(W_alpha2*L2*t(blip_mat2))%*%c(Y-A2*t(blip_mat2)%*%psi_2_lm)/n

eta2_d_alpha2=cbind(eta2_d_alpha2_1,eta2_d_alpha2_2)

U_psi2_adj_lm=U_psi2_lm-(U_psi2_d_alpha2_lm+U_psi2_d_eta2%*%eta2_d_alpha2)%*%
  solve(U_alpha2_d_alpha2)%*%U_alpha2-
  U_psi2_d_eta2%*%solve(U_eta2_d_eta2)%*%U_eta2

meat_2_lm=tcrossprod(U_psi2_adj_lm)/n
bread_2_lm=solve(U_psi2_d_psi2_lm+U_psi2_d_eta2%*%eta2_d_psi2)
var_psi2_lm=bread_2_lm%*%meat_2_lm%*%t(bread_2_lm)/n
sd_psi2_lm=sqrt(diag(var_psi2_lm))
sd_psi2_lm
```

```
##                        L2
## 126.3441420   0.2437377
```

```
#standard error of the estimate of psi_1 from A2
eta1=lm_1$coefficients[-c(1,3)]
R_out1_lm=c(Y+reg_2_lm-A1*t(blip_mat1)%*%psi_1_lm-pre_trt_1*t(blip_mat1)%*%eta1)
U_psi1_lm=matrix(c(R_out1_lm*A1,R_out1_lm*(A1*L1)),nrow=2,ncol=n,byrow = TRUE)
U_psi1_d_psi1_lm=-tcrossprod(t(t(blip_mat1)*A1),blip_mat1)/n
U_psi1_d_eta1=-tcrossprod(t(t(blip_mat1)*(A1*pre_trt_1)),blip_mat1)/n
W1_var_lm=pre_trt_1*(1-pre_trt_1)*A1*c(t(blip_mat1)%*%eta1)
U_psi1_d_alpha1_lm=-tcrossprod(t(t(blip_mat1)*W1_var_lm),trt_mat1)/n

U_eta1=matrix(c(R_out1_lm*pre_trt_1,R_out1_lm*(pre_trt_1*L1)),nrow=2,
              ncol=n,byrow = TRUE)
U_eta1_d_eta1=-tcrossprod(t(t(blip_mat1)*(pre_trt_1^2)),blip_mat1)/n

eta1_mat=tcrossprod(t(t(blip_mat1)*(pre_trt_1^2)),blip_mat1)/n
eta1_vec=t(pre_trt_1*t(blip_mat1))%*%c(Y+reg_2_lm-A1*t(blip_mat1)%*%psi_1_lm)/n

eta1_d_psi1=-solve(eta1_mat)%*%(tcrossprod(t(t(blip_mat1)*(A1*pre_trt_1)),blip_mat1)/n)
W_alpha1=pre_trt_1*(1-pre_trt_1)
```

```
eta1_d_alpha1_1=-solve(eta1_mat)%*%(tcrossprod(t(t(blip_mat1)*(2*pre_trt_1*W_alpha1)),
                                               blip_mat1)/n)%*%solve(eta1_mat)%*%eta1_vec+
  solve(eta1_mat)%*%t(W_alpha1*t(blip_mat1))%*%c(Y+reg_2_lm-A1*t(blip_mat1)%*%
                                                   psi_1_lm)/n

eta1_d_alpha1_2=-solve(eta1_mat)%*%(tcrossprod(t(t(blip_mat1)*(2*pre_trt_1*W_alpha1*L1)),
                                               blip_mat1)/n)%*%solve(eta1_mat)%*%eta1_vec+
  solve(eta1_mat)%*%t(W_alpha1*L1*t(blip_mat1))%*%c(Y+reg_2_lm-A1*t(blip_mat1)%*%
                                                      psi_1_lm)/n

eta1_d_alpha1=cbind(eta1_d_alpha1_1,eta1_d_alpha1_2)

U_psi1_d_psi2_lm=tcrossprod(t(t(blip_mat1)*(A1*(((psi_2_lm[1]+psi_2_lm[2]*L2)>=0)-A2))),
                            blip_mat2)/n
eta1_d_psi2=solve(eta1_mat)%*%
  tcrossprod(t(t(blip_mat1)*(pre_trt_1*(((psi_2_lm[1]+psi_2_lm[2]*L2)>=0)-A2))),
             blip_mat2)/n

U_psi1_adj_lm=U_psi1_lm-(U_psi1_d_alpha1_lm+U_psi1_d_eta1%*%eta1_d_alpha1)%*%
  solve(U_alpha1_d_alpha1)%*%U_alpha1-
  (U_psi1_d_psi2_lm+U_psi1_d_eta1%*%eta1_d_psi2)%*%bread_2_lm%*%U_psi2_adj_lm-
  U_psi1_d_eta1%*%solve(U_eta1_d_eta1)%*%U_eta1

meat_1_lm=tcrossprod(U_psi1_adj_lm)/n
bread_1_lm=solve(U_psi1_d_psi1_lm+U_psi1_d_eta1%*%eta1_d_psi1)

var_psi1_lm=bread_1_lm%*%meat_1_lm%*%t(bread_1_lm)/n
sd_psi1_lm=sqrt(diag(var_psi1_lm))
sd_psi1_lm
```

```
##                      L1
## 127.5384725   0.2864774
```

As the standard errors of the estimates from A1 and A2 are large, the values of $\hat{\psi}_2$ and $\hat{\psi}_1$ from A.1 and A.2 will vary a lot when changing the random seed. The results in Table 6 in the tutorial are obtained under 1000 Monte Carlo replications.

- **A3**: A-learning with the linear treatment-free term $D_j^\top \xi_j$ with $D_1 = (1, L_1)$ and $D_2 = (1, L_1, A_1, L_1 A_1, L_2)$. A3 can be implemented by using 'DTRreg' function as follows:

```
blip.mod=list(~L1,~L2)
treat.mod=list(A1~L1,A2~L2)
tf.mod=list(~L1, ~L1+A1+A1:L1+L2)
res_DTR=DTRreg(Y,blip.mod,treat.mod,tf.mod,data=data,
               method = "gest",var.estim = "sandwich")
psi_dr=unlist(res_DTR$psi)
summary(res_DTR)
```

```
## DTR estimation over  2  stages:
##
##  Blip parameter estimates
## Stage 1 (n = 500)
```

```
##       Estimate Std_Error X95._CI_Lower X95._CI_Upper
## A1    304.4344   26.2047      253.0731      355.7956
## L1:A1  -1.1296    0.0580       -1.2432       -1.0160
## Stage 2 (n = 500)
##       Estimate Std_Error X95._CI_Lower X95._CI_Upper
## A2    677.8338   71.4113      537.8677      817.7999
## L2:A2  -1.9218    0.1256       -2.1680       -1.6756
##
## Warning: possible non-regularity at stages 1, 2 (probs = 0.180, 0.286, respectively)
## Recommended dynamic treatment regimen:
## Stage 1: treat if 304.4344  - 1.1296 L1:A1 > 0
## Stage 2: treat if 677.8338  - 1.9218 L2:A2 > 0
```

- **A4**: regression adjustment for propensity score; i.e., the OLS regression of $\tilde{V}^A_{j+1}$ on $A_j R_j$, $\mathbb{E}\left(A_j|H_j, \hat{\alpha}_j\right) R_j$ and D_j. The estimate of ψ from A4 is given below.

```
lm_2_dr=lm(Y~A2+A2:L2+pre_trt_2+pre_trt_2:L2+L1+A1+A1:L1+L2)
psi_2_lm_dr=lm_2_dr$coefficients[c(2,7)]
reg_2_lm_dr=(((psi_2_lm_dr[1]+psi_2_lm_dr[2]*L2)>=0)-A2)*(psi_2_lm_dr[1]+psi_2_lm_dr[2]*L2)
lm_1_dr=lm((Y+reg_2_lm_dr)~A1+A1:L1+pre_trt_1+pre_trt_1:L1+L1)
psi_1_lm_dr=lm_1_dr$coefficients[c(2,5)]
psi_lm_dr=c(psi_1_lm_dr,psi_2_lm_dr)
psi_lm_dr
```

```
##         A1      A1:L1         A2      A2:L2
## 311.728799  -1.144511 710.697360  -1.982914
```

And the standard errors are calculated as follows.

```
#standard error of the estimate of psi_2 from A4
xi_2_lm=lm_2_dr$coefficients[c(1,4,5,9,6)]
eta2_dr=lm_2_dr$coefficients[c(3,8)]
beta2=c(eta2_dr,xi_2_lm)

lm_psi2_mat=rbind(A2,A2*L2)
trtfre_mat2=rbind(numeric(n)+1,L1,A1,A1*L1,L2)
lm_beta2_mat=rbind(rbind(pre_trt_2,pre_trt_2*L2),trtfre_mat2)

R_out2_lm_dr=c(Y-t(lm_psi2_mat)%*%psi_2_lm_dr-t(lm_beta2_mat)%*%beta2)
U_psi2_lm_dr=matrix(c(R_out2_lm_dr*A2,R_out2_lm_dr*(A2*L2)),nrow=2,ncol=n,byrow = TRUE)
U_psi2_d_psi2_lm_dr=-tcrossprod(t(t(blip_mat2)*A2),blip_mat2)/n
U_psi2_d_beta2=-tcrossprod(lm_psi2_mat,lm_beta2_mat)/n
W2_var_lm_dr=pre_trt_2*(1-pre_trt_2)*A2*c(t(blip_mat2)%*%eta2_dr)
U_psi2_d_alpha2_lm_dr=-tcrossprod(t(t(blip_mat2)*W2_var_lm_dr),trt_mat2)/n

U_beta2=t(t(lm_beta2_mat)*R_out2_lm_dr)
U_beta2_d_beta2=-tcrossprod(lm_beta2_mat,lm_beta2_mat)/n

beta2_mat=tcrossprod(lm_beta2_mat,lm_beta2_mat)/n
beta2_vec=lm_beta2_mat%*%c(Y-A2*t(blip_mat2)%*%psi_2_lm_dr)/n
#solve(beta2_mat)%*%beta2_vec
beta2_d_psi2=-solve(beta2_mat)%*%(tcrossprod(lm_beta2_mat,lm_psi2_mat)/n)
```

```
W_alpha2=pre_trt_2*(1-pre_trt_2)
d_alpha2_1=rbind(rbind(W_alpha2,W_alpha2*L2),matrix(0,ncol=n,nrow=length(xi_2_lm)))
d_alpha2_2=rbind(rbind(W_alpha2*L2,W_alpha2*L2^2),matrix(0,ncol=n,nrow=length(xi_2_lm)))
mat_d_alpha2_1=tcrossprod(d_alpha2_1,lm_beta2_mat)/n
mat_d_alpha2_2=tcrossprod(d_alpha2_2,lm_beta2_mat)/n

beta2_d_alpha2_1=-solve(beta2_mat)%*%(mat_d_alpha2_1+t(mat_d_alpha2_1))%*%
  solve(beta2_mat)%*%beta2_vec+
  solve(beta2_mat)%*%d_alpha2_1%*%c(Y-A2*t(blip_mat2)%*%psi_2_lm_dr)/n

beta2_d_alpha2_2=-solve(beta2_mat)%*%(mat_d_alpha2_2+t(mat_d_alpha2_2))%*%
  solve(beta2_mat)%*%beta2_vec+
  solve(beta2_mat)%*%d_alpha2_2%*%c(Y-A2*t(blip_mat2)%*%psi_2_lm_dr)/n

beta2_d_alpha2=cbind(beta2_d_alpha2_1,beta2_d_alpha2_2)

U_psi2_adj_lm_dr=U_psi2_lm_dr-(U_psi2_d_alpha2_lm_dr+U_psi2_d_beta2%*%beta2_d_alpha2)%*%
  solve(U_alpha2_d_alpha2)%*%U_alpha2-
  U_psi2_d_beta2%*%solve(U_beta2_d_beta2)%*%U_beta2

meat_2_lm_dr=tcrossprod(U_psi2_adj_lm_dr)/n
bread_2_lm_dr=solve(U_psi2_d_psi2_lm_dr+U_psi2_d_beta2%*%beta2_d_psi2)
var_psi2_lm_dr=bread_2_lm_dr%*%meat_2_lm_dr%*%t(bread_2_lm_dr)/n
sd_psi2_lm_dr=sqrt(diag(var_psi2_lm_dr))
sd_psi2_lm_dr
```

```
##                      L2
## 23.36653779  0.04485942
```

```
#standard error of the estimate of psi_1 from A4
xi_1_lm=lm_1_dr$coefficients[c(1,4)]
eta1_dr=lm_1_dr$coefficients[c(3,6)]
beta1=c(eta1_dr,xi_1_lm)

lm_psi1_mat=rbind(A1,A1*L1)
trtfre_mat1=rbind(numeric(n)+1,L1)
lm_beta1_mat=rbind(rbind(pre_trt_1,pre_trt_1*L1),trtfre_mat1)

R_out1_lm_dr=c(Y+reg_2_lm_dr-t(lm_psi1_mat)%*%psi_1_lm_dr-t(lm_beta1_mat)%*%beta1)
U_psi1_lm_dr=matrix(c(R_out1_lm_dr*A1,R_out1_lm_dr*(A1*L1)),nrow=2,ncol=n,byrow = TRUE)
U_psi1_d_psi1_lm_dr=-tcrossprod(t(t(blip_mat1)*A1),blip_mat1)/n
U_psi1_d_beta1=-tcrossprod(lm_psi1_mat,lm_beta1_mat)/n
W1_var_lm_dr=pre_trt_1*(1-pre_trt_1)*A1*c(t(blip_mat1)%*%eta1_dr)
U_psi1_d_alpha1_lm_dr=-tcrossprod(t(t(blip_mat1)*W1_var_lm_dr),trt_mat1)/n

U_beta1=t(t(lm_beta1_mat)*R_out1_lm_dr)
U_beta1_d_beta1=-tcrossprod(lm_beta1_mat,lm_beta1_mat)/n

beta1_mat=tcrossprod(lm_beta1_mat,lm_beta1_mat)/n
beta1_vec=lm_beta1_mat%*%c(Y+reg_2_lm_dr-A1*t(blip_mat1)%*%psi_1_lm_dr)/n
beta1_d_psi1=-solve(beta1_mat)%*%(tcrossprod(lm_beta1_mat,lm_psi1_mat)/n)

W_alpha1=pre_trt_1*(1-pre_trt_1)
```

```r
d_alpha1_1=rbind(rbind(W_alpha1,W_alpha1*L1),matrix(0,ncol=n,nrow=length(xi_1_lm)))
d_alpha1_2=rbind(rbind(W_alpha1*L1,W_alpha1*L1^2),matrix(0,ncol=n,nrow=length(xi_1_lm)))
mat_d_alpha1_1=tcrossprod(d_alpha1_1,lm_beta1_mat)/n
mat_d_alpha1_2=tcrossprod(d_alpha1_2,lm_beta1_mat)/n

beta1_d_alpha1_1=-solve(beta1_mat)%*%(mat_d_alpha1_1+t(mat_d_alpha1_1))%*%
  solve(beta1_mat)%*%beta1_vec+
  solve(beta1_mat)%*%d_alpha1_1%*%c(Y+reg_2_lm_dr-A1*t(blip_mat1)%*%psi_1_lm_dr)/n
beta1_d_alpha1_2=-solve(beta1_mat)%*%(mat_d_alpha1_2+t(mat_d_alpha1_2))%*%
  solve(beta1_mat)%*%beta1_vec+
  solve(beta1_mat)%*%d_alpha1_2%*%c(Y+reg_2_lm_dr-A1*t(blip_mat1)%*%psi_1_lm_dr)/n
beta1_d_alpha1=cbind(beta1_d_alpha1_1,beta1_d_alpha1_2)

U_psi1_d_psi2_lm_dr=tcrossprod(t(t(blip_mat1)*(A1*(((psi_2_lm_dr[1]+
                                                     psi_2_lm_dr[2]*L2)>=0)-A2))),
                               blip_mat2)/n
beta1_d_psi2=solve(beta1_mat)%*%tcrossprod(t(t(lm_beta1_mat)*
                                    (((psi_2_lm_dr[1]+psi_2_lm_dr[2]*L2)>=0)-A2)),
                                    blip_mat2)/n

U_psi1_adj_lm_dr=U_psi1_lm_dr-(U_psi1_d_alpha1_lm_dr+U_psi1_d_beta1%*%beta1_d_alpha1)%*%
  solve(U_alpha1_d_alpha1)%*%U_alpha1-
  U_psi1_d_beta1%*%solve(U_beta1_d_beta1)%*%U_beta1-
  (U_psi1_d_psi2_lm_dr+U_psi1_d_beta1%*%beta1_d_psi2)%*%bread_2_lm_dr%*%U_psi2_adj_lm_dr

meat_1_lm_dr=tcrossprod(U_psi1_adj_lm_dr)/n
bread_1_lm_dr=solve(U_psi1_d_psi1_lm_dr+U_psi1_d_beta1%*%beta1_d_psi1)
var_psi1_lm_dr=bread_1_lm_dr%*%meat_1_lm_dr%*%t(bread_1_lm_dr)/n
sd_psi1_lm_dr=sqrt(diag(var_psi1_lm_dr))
sd_psi1_lm_dr
```

```
##                     L1
## 20.73389017  0.04633306
```

- **dWOLS**: dynamic weighted OLS developed by (Wallace and Moodie 2015), which is included in the package.

```r
res_DTR_w=DTRreg(Y, blip.mod, treat.mod,tf.mod,data=data,method = "dwols",
                 var.estim = "bootstrap")
psi_wols=unlist(res_DTR_w$psi)
summary(res_DTR_w)
```

```
## DTR estimation over  2  stages:
##
##  Blip parameter estimates
## Stage 1 (n = 500)
##       Estimate Std_Error X95._CI_Lower X95._CI_Upper
## A1    304.1972   27.9649      249.3860      359.0085
## L1:A1  -1.1291    0.0598       -1.2463       -1.0119
## Stage 2 (n = 500)
##       Estimate Std_Error X95._CI_Lower X95._CI_Upper
## A2    675.3710   63.7013      550.5165      800.2255
```

```
## L2:A2  -1.9191    0.1081       -2.1308       -1.7073
##
## Warning: possible non-regularity at stages 1, 2 (probs = 0.200, 0.248, respectively)
## Recommended dynamic treatment regimen:
## Stage 1: treat if 304.1972  - 1.1291 L1:A1 > 0
## Stage 2: treat if 675.3710  - 1.9191 L2:A2 > 0
```

Causal Tree (CT)

```
pro_trt2=mean(A2)
ipt2=ifelse(A2==1,pro_trt2/pre_trt_2,(1-pro_trt2)/(1-pre_trt_2))

tree2ipw=causalTree(Y~L2, treatment = A2, data=data, weights = ipt2,
                    split.Rule = "CT", cv.option = "CT", split.Honest = T,
                    cv.Honest = T, split.Bucket = T,
                    xval = 5, cp = 0, minsize = 10,bucketMax = 20)

rpart.plot(tree2ipw)
```

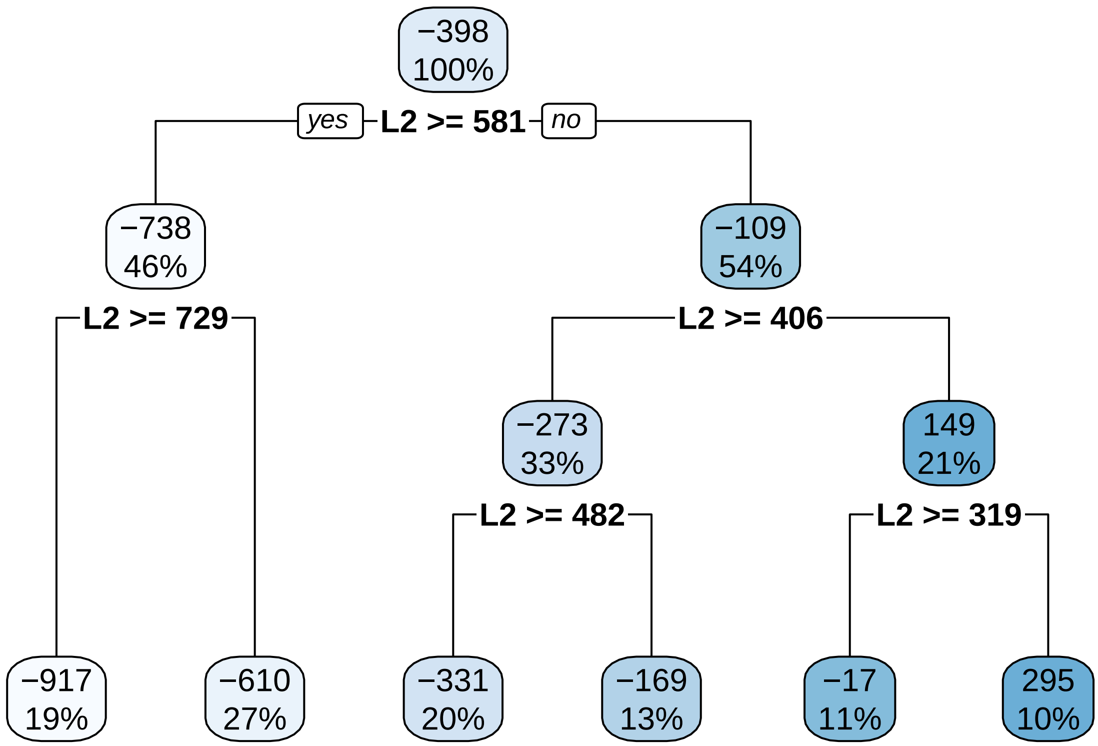


```
contra2=predict(tree2ipw)

Y_opt2=data$Y+((contra2>0)-data$A2)*contra2
data$Y_opt2=Y_opt2
```

```
pro_trt1=mean(A1)
ipt1=ifelse(A1==1,pro_trt1/pre_trt_1,(1-pro_trt1)/(1-pre_trt_1))
tree1ipw=causalTree(Y_opt2~L1, treatment = A1, data=data,weights = ipt1,
                    split.Rule = "CT", cv.option = "CT", split.Honest = T,
                    cv.Honest = T, split.Bucket = T,
                    xval = 5, cp = 0,minsize=5,bucketMax = 20)
rpart.plot(tree1ipw)
```

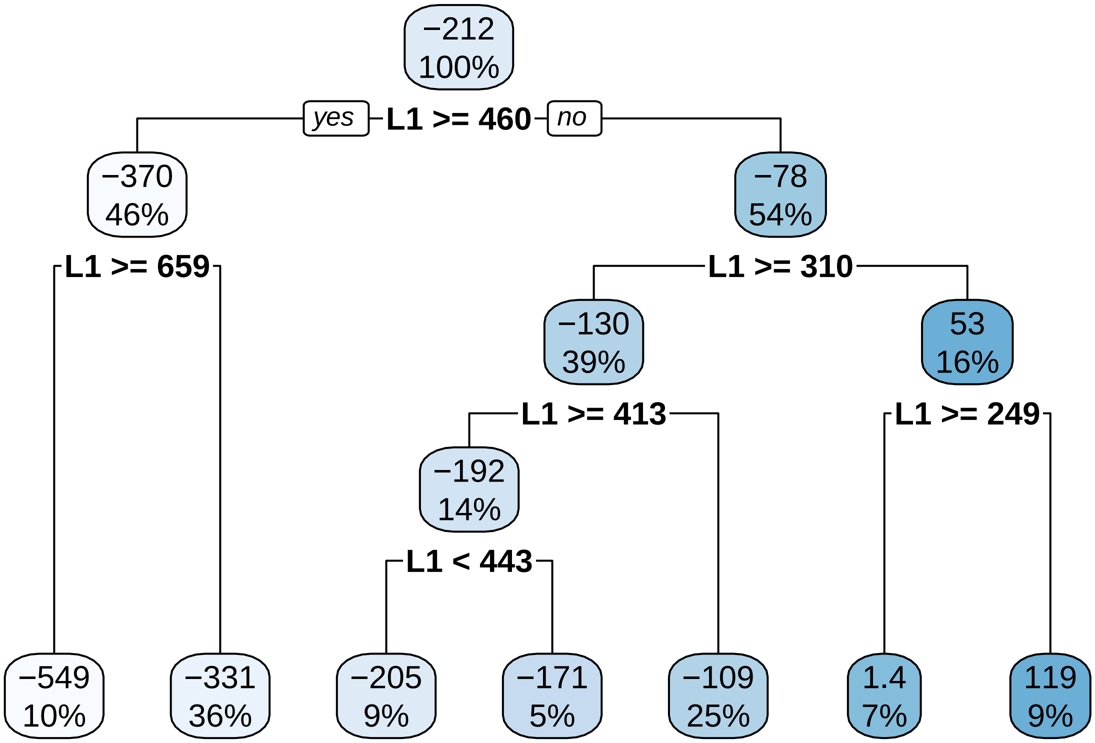

Note that *weights* specified in 'causalTree' is to correct for variations in propensity scores inside the leaf when the leaves are not small enough. Besides the inverse probability weighting used above, $|A_j - \mathbb{E}[A_j|H_j]|$ can also be used as the weights to correct for bias due to confounding.

IPWE

The objective function IPWE(ψ) is defined as follows.

```
est.ipw=function(data){
  n=dim(data)[1]
  #### Estimate propensity score
  trt_1=glm(data$A1~data$L1,family = binomial)
  trt_2=glm(data$A2~data$L2,family = binomial)
  pre_trt_2=predict(trt_2,type = "response")
  pre_trt_1=predict(trt_1,type = "response")

  blip_mat2=rbind(numeric(n)+1,data$L2)
```

```
  blip_mat1=rbind(numeric(n)+1,data$L1)


  objfun_ipw=function(par){

    psi_1_ipw=c(par[1],-1)
    #psi_1_ipw=par[1:2]
    psi_2_ipw=c(par[2],-1)
    #psi_2_ipw=par[3:4]

    ###indicator: whether the observed treatment sequence is consistent with
    ### the treatment regime indexed by psi
    contra1_ipw=c(t(blip_mat1)%*%psi_1_ipw)
    contra2_ipw=c(t(blip_mat2)%*%psi_2_ipw)
    consis_categor=as.numeric(data$A1==as.numeric(contra1_ipw>0))+
      as.numeric(data$A2==as.numeric(contra2_ipw>0))
    consis_ind=as.numeric(consis_categor==2)
    consis_pro=ifelse(contra1_ipw>0,pre_trt_1,1-pre_trt_1)*
      ifelse(contra2_ipw>0,pre_trt_2,1-pre_trt_2)
    marmean_poten_ipw=sum(data$Y[consis_ind==1]/consis_pro[consis_ind==1])/n
    return(marmean_poten_ipw)
  }

  low_bou=c(0,0)
  upp_bou=c(max(data$L1),max(data$L2))


  res_ipw=tryCatch(genoud(objfun_ipw,max=TRUE,nvars =2,pop.size = 1000,
                          starting.values = c(mean(data$L1),mean(data$L2)),
                          Domains = cbind(low_bou,upp_bou),
                          optim.method ="Nelder-Mead",print.level=0),
                   warning=function(x) return(NULL))

  if(is.null(res_ipw)){
    return(list(thresh_1_aipw=0,thresh_2_aipw=0))
  }

  par_ipw=res_ipw$par

  thresh_1_ipw=par_ipw[1]
  thresh_2_ipw=par_ipw[2]


  return(list(thresh_1_ipw=thresh_1_ipw,thresh_2_ipw=thresh_2_ipw))

}
```

And IPWE returns the value of parameters which maximizes IPWE(ψ) over the feasible set.

```
psi_ipw=unlist(est.ipw(data))
psi_ipw
```

```
## thresh_1_ipw thresh_2_ipw
##     353.9142     423.8756
```

AIPWE

The objective function AIPWE(ψ) is defined as follows.

```
est.aipw=function(data){
   n=dim(data)[1]
   #### Estimate propensity score
   trt_1=glm(data$A1~data$L1,family = binomial)
   trt_2=glm(data$A2~data$L2,family = binomial)
   pre_trt_2=predict(trt_2,type = "response")
   pre_trt_1=predict(trt_1,type = "response")

   blip_mat2=rbind(numeric(n)+1,data$L2)
   blip_mat1=rbind(numeric(n)+1,data$L1)

   ### AIPW
   objfun_aipw=function(par){

      psi_1_aipw=c(par[1],-1)
      psi_2_aipw=c(par[2],-1)

      contra1_aipw=c(t(blip_mat1)%*%psi_1_aipw)
      contra2_aipw=c(t(blip_mat2)%*%psi_2_aipw)

      ### Construct a categorical variable taking values 1 if failing to match in stage 1,
      #### or 2 if being matched in stage 1 but failing to match in stage 2,
      #### or 3 if being fully matched in both stages.
      consis_categor_aipw=ifelse(data$A1!=as.numeric(contra1_aipw>0),1,
                                 ifelse(data$A2!=as.numeric(contra2_aipw>0),2,3))
      ###this is a simple case because pro(A2|L2,...) does not depend on A1
      ###hazard: fail to match at the kth stage given it matches through k-1 stages.
      haz1=ifelse(contra1_aipw>0,1-pre_trt_1,pre_trt_1)
      haz2=numeric(n)+1
      haz2[consis_categor_aipw>1]=ifelse(contra2_aipw[consis_categor_aipw>1]>0,
                                         1-pre_trt_2[consis_categor_aipw>1],
                                         pre_trt_2[consis_categor_aipw>1])
      ####prob_k=prob(consis_categor_aipw>k)
      prob_1=1-haz1
      prob_2=numeric(n)+1
      prob_2[consis_categor_aipw>=2]=(1-haz1[consis_categor_aipw>=2])*
        (1-haz2[consis_categor_aipw>=2])


      ####augment terms
      lm_2_q=lm(Y~A2+A2:L2+L1+A1+A1:L1+L2,data=data)
      trtfre_mat2_q=rbind(numeric(n)+1,data$L1,data$A1,data$L2,data$A1*data$L1)
      psi_2_q=lm_2_q$coefficients[c(2,6)]
      beta_2_q=lm_2_q$coefficients[-c(2,6)]
      V_2=(c(t(blip_mat2)%*%psi_2_q)>0)*c(t(blip_mat2)%*%psi_2_q)+
        t(trtfre_mat2_q)%*%beta_2_q
      lm_1_q=lm(V_2~A1+A1:L1+L1,data=data)
      newdata_1=data.frame(L1=data$L1,A1=as.numeric(contra1_aipw>0))
      newdata_2=data.frame(L1=data$L1,A1=as.numeric(contra1_aipw>0),
                           L2=data$L2,A2=as.numeric(contra2_aipw>0))
```

```
    aug1=(as.numeric(consis_categor_aipw==1)-haz1)/prob_1*predict(lm_1_q,newdata_1)
    aug2=numeric(n)
    aug2[consis_categor_aipw>1]=
      (as.numeric(consis_categor_aipw[consis_categor_aipw>1]==2)-
         haz2[consis_categor_aipw>1])/prob_2[consis_categor_aipw>1]*
      predict(lm_2_q,newdata_2[consis_categor_aipw>1,])


    marmean_poten_aipw=sum(data$Y[consis_categor_aipw==3]/
                             prob_2[consis_categor_aipw==3])/n+sum(aug1+aug2)/n

    return(marmean_poten_aipw)
  }

  low_bou=c(0,0)
  upp_bou=c(max(data$L1),max(data$L2))


  res_aipw=tryCatch(genoud(objfun_aipw,max=TRUE,nvars = 2,pop.size = 1000,
                           starting.values = c(mean(data$L1),mean(data$L2)),
                           Domains = cbind(low_bou,upp_bou),
                           optim.method ="Nelder-Mead",print.level=0),
                    warning=function(x) return(NULL))

  if(is.null(res_aipw)){
    return(list(thresh_1_aipw=0,thresh_2_aipw=0))
  }

  par_aipw=res_aipw$par

  thresh_1_aipw=par_aipw[1]
  thresh_2_aipw=par_aipw[2]

  return(list(thresh_1_aipw=thresh_1_aipw,thresh_2_aipw=thresh_2_aipw))

}
```

And AIPWE returns the value of parameters which maximizes AIPWE(ψ) over the feasible set.

```
psi_aipw=unlist(est.aipw(data))
psi_aipw
```

```
## thresh_1_aipw thresh_2_aipw
##      267.6990      356.1058
```

Assess the performance of $\hat{d}^{\mathbf{opt}}$ via $\mathbb{E}[Y^*(\hat{d}^{\mathbf{opt}})]$

For a given estimate of d^{opt}, say, $\hat{d}^{\text{opt}}$ which is indexed by $\hat{\psi}$, the achieved average value of the outcome $\mathbb{E}[Y^*(\hat{d}^{\text{opt}})]$ is calculated as follows.

```r
mean.opt=function(par,B){#parameters should be passed in order (psi_10,psi_11,psi_20,psi_21).
   L1_sam=rnorm(B,mean=450,sd=150)
   L2_sam=rnorm(B,mean=1.25*L1_sam,sd=60)
   reg1=((L1_sam<=250)-(par[1]+par[2]*L1_sam>0))*(250-L1_sam)
   reg2=((L2_sam<=360)-(par[3]+par[4]*L2_sam>0))*(720-2*L2_sam)
   Y=rnorm(B,mean=400+1.6*L1_sam,sd=60)-reg1-reg2
   return(mean(Y))
}
```

```r
B=10000
mean.opt(c(250,-1,720,-2),B) ##True
```

```
## [1] 1119.978
```

```r
mean.opt(c(psi_ipw[1],-1,psi_ipw[2],-1),B) ##IPWE
```

```
## [1] 1106.504
```

```r
mean.opt(c(psi_aipw[1],-1,psi_aipw[2],-1),B) ##AIPWE
```

```
## [1] 1121.183
```

```r
mean.opt(psi_q,B) ##Q-learning
```

```
## [1] 1114.974
```

```r
mean.opt(psi_dr,B) ##A3
```

```
## [1] 1117.423
```

The achieved average value of the outcome $\mathbb{E}[Y^*(\hat{d}^{\text{opt}})]$ under CT is calculated as follows.

```r
   mean.opt.tree=function(tree1,tree2,B){
      L1_sam=rnorm(B,mean=450,sd=150)
      L2_sam=rnorm(B,mean=1.25*L1_sam,sd=60)
      contra1_sam=predict(tree1,newdata = data.frame(L1=L1_sam))
      contra2_sam=predict(tree2,newdata = data.frame(L2=L2_sam))

      reg1=((L1_sam<=250)-as.numeric(contra1_sam>0))*(250-L1_sam)
      reg2=((L2_sam<=360)-as.numeric(contra2_sam>0))*(720-2*L2_sam)
      Y=rnorm(B,mean=400+1.6*L1_sam,sd=60)-reg1-reg2
      #return(c(mean(400+1.6*L1_sam-reg1-reg2),mean(Y)))
      return(mean(Y))
   }
```

```r
mean.opt.tree(tree1ipw,tree2ipw,B) ##causal tree
```

```
## [1] 1119.955
```

2. Example code for simulation studies in Section 4.2

For a given $\hat{d}^{\text{opt}}$ determined by parameters (e.g., $\hat{d}^{\text{opt}}$ obtained from Q-learning, A-learning), we calculate $\mathbb{E}[Y^*(\hat{d}^{\text{opt}})]$ as follows:

```
mean.opt=function(par,B){## par is a list of length K.
  ###stage 1
  W_sam=rtnorm(B,mu=45,sd=10,lb=10,ub=Inf) ##e.g.,age
  L11_sam=rtnorm(B,mu=20,sd=5,lb=0,ub=Inf)##the higher, the worse.
  L12_sam=rtnorm(B,mu=10,sd=3,lb=0,ub=Inf)
  blip_mat1=cbind(numeric(B)+1,W_sam,L11_sam,L12_sam)
  contra1=c(blip_mat1%*%par[[1]])

  ###stage 2
  L21_sam=rtnorm(1,mu=1.25*L11_sam-2*as.numeric(contra1>0),
                 sd=5,lb=0,ub=Inf)
  L22_sam=rtnorm(1,mu=L12_sam-as.numeric(contra1>0),sd=3,
                 lb=0,ub=Inf)
  blip_mat2=cbind(blip_mat1,as.numeric(contra1>0),L21_sam,L22_sam)
  contra2=c(blip_mat2%*%par[[2]])

  ###stage 3
  L31_sam=rtnorm(1,mu=L21_sam-2*(as.numeric(contra2>0)+as.numeric(contra1>0)),
                 sd=5,lb=0,ub=Inf)
  L32_sam=rtnorm(1,mu=L22_sam-as.numeric(contra2>0),sd=3,
                 lb=0,ub=Inf)
  blip_mat3=cbind(blip_mat2,as.numeric(contra2>0),L31_sam,L32_sam)
  contra3=c(blip_mat3%*%par[[3]])


  reg1=(as.numeric((L11_sam>30)|(L12_sam>12))-as.numeric(contra1>0))^2*
    (abs(L11_sam-30)+abs(L12_sam-12))/2
  reg2=(as.numeric((L21_sam>25)|(L22_sam>10))-as.numeric(contra2>0))^2*
    (abs(L21_sam-25)+abs(L22_sam-10))
  reg3=(as.numeric((L31_sam+L32_sam)>35)-as.numeric(contra3>0))^2*log(W_sam)*2

  Y=rnorm(B,mean=100,sd=10)-reg1-reg2-reg3
  return(mean(Y))
}
```

For a given $\hat{d}^{\text{opt}}$ from CT, we calculate $\mathbb{E}[Y^*(\hat{d}^{\text{opt}})]$ as follows:

```
mean.opt.tree=function(tree1,tree2,tree3,B){
  ###stage 1
  W_sam=rtnorm(B,mu=45,sd=10,lb=10,ub=Inf) ##e.g.,age
  L11_sam=rtnorm(B,mu=20,sd=5,lb=0,ub=Inf)##the higher, the worse.
  L12_sam=rtnorm(B,mu=10,sd=3,lb=0,ub=Inf)
  contra1=predict(tree1,newdata = data.frame(W=W_sam,L11=L11_sam,L12=L12_sam))

  ###stage 2
  L21_sam=rtnorm(1,mu=1.25*L11_sam-2*as.numeric(contra1>0),
                 sd=5,lb=0,ub=Inf)
  L22_sam=rtnorm(1,mu=L12_sam-as.numeric(contra1>0),sd=3,
```

```
                 lb=0,ub=Inf)
  contra2=predict(tree2,newdata = data.frame(W=W_sam,L21=L21_sam,L22=L22_sam,
                                             A1=as.numeric(contra1>0),
                                             L11=L11_sam,L12=L12_sam))

  ###stage 3
  L31_sam=rtnorm(1,mu=L21_sam-2*(as.numeric(contra2>0)+as.numeric(contra1>0)),
                 sd=5,lb=0,ub=Inf)
  L32_sam=rtnorm(1,mu=L22_sam-as.numeric(contra2>0),sd=3,
                 lb=0,ub=Inf)
  contra3=predict(tree3,newdata=data.frame(W=W_sam,L31=L31_sam,L32=L32_sam,
                        A2=as.numeric(contra2>0),
                        W=W_sam,L21=L21_sam,L22=L22_sam,
                        A1=as.numeric(contra1>0),L11=L11_sam,L12=L12_sam))


  reg1=(as.numeric((L11_sam>30)|(L12_sam>12))-as.numeric(contra1>0))^2*
    (abs(L11_sam-30)+abs(L12_sam-12))/2
  reg2=(as.numeric((L21_sam>25)|(L22_sam>10))-as.numeric(contra2>0))^2*
    (abs(L21_sam-25)+abs(L22_sam-10))
  reg3=(as.numeric((L31_sam+L32_sam)>35)-as.numeric(contra3>0))^2*log(W_sam)*2

  Y=rnorm(B,mean=100,sd=10)-reg1-reg2-reg3
  return(mean(Y))
}
```

For a given $\hat{d}^{\text{opt}}$ from OWL, we calculate $\mathbb{E}[Y^*(\hat{d}^{\text{opt}})]$ as follows:

```
mean.opt.owl=function(owl,kernel,B){
  ###stage 1
  W_sam=rtnorm(B,mu=45,sd=10,lb=10,ub=Inf) ##e.g.,age
  L11_sam=rtnorm(B,mu=20,sd=5,lb=0,ub=Inf)##the higher, the worse.
  L12_sam=rtnorm(B,mu=10,sd=3,lb=0,ub=Inf)
  H1_sam=scale(cbind(W_sam,L11_sam,L12_sam))

  if(kernel=='linear'){
    fit1=owl$stage1$beta0+H1_sam%*%owl$stage1$beta
  }else{
    rbf  = rbfdot(sigma = owl$stage1$sigma)
    fit1=owl$stage1$beta0+
      kernelMatrix(rbf,H1_sam,owl$stage1$H)%*%c(owl$stage1$alpha1)
  }
  ###stage 2
  L21_sam=rtnorm(1,mu=1.25*L11_sam-2*as.numeric(fit1>0),
                 sd=5,lb=0,ub=Inf)
  L22_sam=rtnorm(1,mu=L12_sam-as.numeric(fit1>0),sd=3,
                 lb=0,ub=Inf)

  H2_sam=cbind(H1_sam,2*as.numeric(fit1>0)-1,scale(cbind(L21_sam,L22_sam)))

  if(kernel=='linear'){
    fit2=owl$stage2$beta0+H2_sam%*%owl$stage2$beta
  }else{
```

```
    rbf = rbfdot(sigma = owl$stage2$sigma)
    fit2=owl$stage2$beta0+
      kernelMatrix(rbf,H2_sam,owl$stage2$H)%*%c(owl$stage2$alpha1)
  }
  ###stage 3
  L31_sam=rtnorm(1,mu=L21_sam-2*(as.numeric(fit2>0)+as.numeric(fit1>0)),
                 sd=5,lb=0,ub=Inf)
  L32_sam=rtnorm(1,mu=L22_sam-as.numeric(fit2>0),sd=3,
                 lb=0,ub=Inf)

  H3_sam=cbind(H2_sam,2*as.numeric(fit2>0)-1,scale(cbind(L31_sam,L32_sam)))

  if(kernel=='linear'){
    fit3=owl$stage3$beta0+H3_sam%*%owl$stage3$beta
  }else{
    rbf = rbfdot(sigma = owl$stage3$sigma)
    fit3=owl$stage3$beta0+
      kernelMatrix(rbf,H3_sam,owl$stage3$H)%*%c(owl$stage3$alpha1)
  }

  reg1=(as.numeric((L11_sam>30)|(L12_sam>12))-as.numeric(fit1>0))^2*
    (abs(L11_sam-30)+abs(L12_sam-12))/2
  reg2=(as.numeric((L21_sam>25)|(L22_sam>10))-as.numeric(fit2>0))^2*
    (abs(L21_sam-25)+abs(L22_sam-10))
  reg3=(as.numeric((L31_sam+L32_sam)>35)-as.numeric(fit3>0))^2*log(W_sam)*2
  Y=rnorm(B,mean=100,sd=10)-reg1-reg2-reg3
  return(mean(Y))
}
```

The implementation details of estimating optimal DTRs in case 2 from the specific methods, Q-learning, A-learning, CT and OWLs (O1,O2 and O3), as well as assessing their performances are given below:

```
sim.case2=function(s,n_tra,n_tes,B){
  set.seed(s)
  print(s)
  ##Generate data.
  n=n_tra+n_tes
  ###stage 1
  W=rtnorm(n,mu=45,sd=10,lb=10,ub=Inf) ##e.g.,age
  L11=rtnorm(n,mu=20,sd=5,lb=0,ub=Inf)##the higher, the worse.
  L12=rtnorm(n,mu=10,sd=3,lb=0,ub=Inf)
  A1=rbinom(n,1,expit(-3+0.1*W))

  ####stage 2
  L21=rtnorm(1,mu=1.25*L11-2*A1,sd=5,lb=0,ub=Inf)
  L22=rtnorm(1,mu=L12-A1,sd=3,lb=0,ub=Inf)
  A2=rbinom(n,1,expit(-1+0.04*(L21+L22)))

  ###stage 3
  L31=rtnorm(1,mu=L21-2*(A2+A1),sd=5,lb=0,ub=Inf)
  L32=rtnorm(1,mu=L22-A2,sd=3,lb=0,ub=Inf)
  A3=rbinom(n,1,expit(-2+0.1*L31))
  ###outcome:the higher, the better.
```

```
Y=rnorm(n,mean=100,sd=10)-
  (as.numeric((L11>30)|(L12>12))-A1)^2*(abs(L11-30)+abs(L12-12))/2-
  (as.numeric((L21>25)|(L22>10))-A2)^2*(abs(L21-25)+abs(L22-10))-
  (as.numeric((L31+L32)>35)-A3)^2*log(W)*2


 data_all=data.frame(W=W,L11=L11,L12=L12,A1=A1,
                     L21=L21,L22=L22,A2=A2,
                     L31=L31,L32=L32,A3=A3,Y=Y)
 data_tra=data_all[1:n_tra,]
 data_tes=data_all[-c(1:n_tra),]

 ######treatment model estimated from the training set
 trt_1=glm(A1~L11+L12+W,family = binomial,data = data_tra)
 trt_2=glm(A2~L21+L22+W,family = binomial,data = data_tra)
 trt_3=glm(A3~L31+L32+W,family = binomial,data = data_tra)

 pre_trt_1=predict(trt_1,type = "response")
 pre_trt_2=predict(trt_2,type = "response")
 pre_trt_3=predict(trt_3,type = "response")

 #####################Causal Tree
 ####################Build the Tree based on training set
 pro_trt3=mean(data_tra$A3)
 ipt3=ifelse(data_tra$A3==1,pro_trt3/pre_trt_3,(1-pro_trt3)/(1-pre_trt_3))
 tree3=causalTree(Y~W+L31+L32+A2+L21+L22+L11+L12+A1,treatment = A3,
                  data=data_tra,weights=ipt3,
                  split.Rule = "CT", cv.option = "CT", split.Honest = T,
                  cv.Honest =T,split.Bucket = T,
                  xval = 5, cp = 0, minsize = 30,bucketMax = 20)

 #rpart.plot(tree3)
 contra3_tree=predict(tree3)
 Y_opt3=data_tra$Y+((contra3_tree>0)-data_tra$A3)*contra3_tree
 data_tra$Y_opt3=Y_opt3
 pro_trt2=mean(data_tra$A2)
 ipt2=ifelse(data_tra$A2==1,pro_trt2/pre_trt_2,(1-pro_trt2)/(1-pre_trt_2))
 tree2=causalTree(Y_opt3~W+L21+L22+L11+L12+A1, treatment = A2, data=data_tra,
                  weights = ipt2, split.Rule = "CT", cv.option = "CT",
                  split.Honest = T,cv.Honest = T,split.Bucket = T,
                  xval = 5, cp = 0,minsize=30,bucketMax = 20)

 contra2_tree=predict(tree2)

 Y_opt2=data_tra$Y_opt3+((contra2_tree>0)-data_tra$A2)*contra2_tree
 data_tra$Y_opt2=Y_opt2
 pro_trt1=mean(data_tra$A1)
 ipt1=ifelse(data_tra$A1==1,pro_trt1/pre_trt_1,(1-pro_trt1)/(1-pre_trt_1))
 tree1=causalTree(Y_opt2~W+L11+L12, treatment = A1, data=data_tra,
                  weights = ipt1, split.Rule = "CT", cv.option = "CT",
                  split.Honest = T, cv.Honest = T,split.Bucket = T,
                  xval = 5, cp = 0,minsize=30,bucketMax = 20)
```

```r
 mean_opt_tree=mean.opt.tree(tree1,tree2,tree3,B)

#########################A-learning
 blip.mod=list(~W+L11+L12,~W+L11+L12+A1+L21+L22,~W+L11+L12+A1+L21+L22+A2+L31+L32)
 treat.mod=list(A1~L11+L12+W,A2~L21+L22+W,A3~L31+L32+W)
 tf.mod=list(~W+L11+L12, ~W+L11+L12+A1+A1:L11+A1:L12+L21+L22,
             ~W+L11+L12+A1+A1:L11+A1:L12+L21+L22+A2+A2:L21+A2:L22+L31+L32)

 res_dr=suppressMessages(DTRreg(data_tra$Y, blip.mod,treat.mod,tf.mod,data=data_tra,
                                method = "gest",var.estim = "sandwich"))
 psi_dr=res_dr$psi## a list
 mean_opt_dr=mean.opt(psi_dr,B)

 #########################Q-learning
 res_q=suppressMessages(DTRreg(data_tra$Y, blip.mod, treat.mod,tf.mod,data=data_tra,
                               method ="qlearn",var.estim = "none"))
 psi_q=res_q$psi
 mean_opt_q=mean.opt(psi_q,B)

 ######################OWL############
 ###recode the treatment to 1/-1
 A3_new=2*data_tra$A3-1
 A2_new=2*data_tra$A2-1
 A1_new=2*data_tra$A1-1

 H1=scale(cbind(data_tra$W,data_tra$L11,data_tra$L12))
 H2=cbind(H1,A1_new,scale(cbind(data_tra$L21,data_tra$L22)))
 H3=cbind(H2,A2_new,scale(cbind(data_tra$L31,data_tra$L32)))

 ##########O1#############
 res_owl_lin=owl(H=list(H1,H2,H3), AA=list(A1_new,A2_new,A3_new),
                 RR=list(rep(0, n_tra),rep(0, n_tra),data_tra$Y),
                 n=length(A3_new), K=3, pi='estimated',
                 res.lasso=TRUE, loss='hinge', kernel='linear',c=2^(-2:4),
                 augment=FALSE)

if(is.null(res_owl_lin$stage1$beta0)){
  return(list(s=s,Tree=rep(NA,5),Alearn=rep(NA,5),Qlearn=rep(NA,5),
            owl_lin=rep(NA,5),owl_lin_aug=rep(NA,5),owl_rbf=rep(NA,5)))
}

if(is.na(res_owl_lin$stage1$beta0)){
  return(list(s=s,Tree=rep(NA,5),Alearn=rep(NA,5),Qlearn=rep(NA,5),
            owl_lin=rep(NA,5),owl_lin_aug=rep(NA,5),owl_rbf=rep(NA,5)))
}

mean_opt_owl_lin=mean.opt.owl(res_owl_lin,'linear',B)

##########O2#############
res_owl_lin_aug=owl(H=list(H1,H2,H3), AA=list(A1_new,A2_new,A3_new),
                    RR=list(rep(0, n_tra),rep(0, n_tra),data_tra$Y),
                    n=length(A3_new), K=3, pi='estimated',
                    res.lasso=TRUE, loss='hinge', kernel='linear',c=2^(-2:4),
```

```
                    augment=TRUE)

if(is.null(res_owl_lin_aug$stage1$beta0)){
  return(list(s=s,Tree=rep(NA,5),Alearn=rep(NA,5),Qlearn=rep(NA,5),
              owl_lin=rep(NA,5),owl_lin_aug=rep(NA,5),owl_rbf=rep(NA,5)))
}

if(is.na(res_owl_lin_aug$stage1$beta0)){
      return(list(s=s,Tree=rep(NA,5),Alearn=rep(NA,5),Qlearn=rep(NA,5),
              owl_lin=rep(NA,5),owl_lin_aug=rep(NA,5),owl_rbf=rep(NA,5)))
}

mean_opt_owl_lin_aug=mean.opt.owl(res_owl_lin_aug,'linear',B)

############O3#################
res_owl_rbf_aug=owl(H=list(H1,H2,H3), AA=list(A1_new,A2_new,A3_new),
                    RR=list(rep(0, n_tra),rep(0, n_tra),data_tra$Y),
                    n=length(A3_new), K=3, pi='estimated',
                    res.lasso=TRUE, loss='hinge', kernel='rbf',c=2^(-2:4),
                    sigma=c(0.05,0.1,0.5,1,2),
                    augment=TRUE)

if(is.null(res_owl_rbf_aug$stage1$beta0)){
  return(list(s=s,Tree=rep(NA,5),Alearn=rep(NA,5),Qlearn=rep(NA,5),
              owl_lin=rep(NA,5),owl_lin_aug=rep(NA,5),owl_rbf=rep(NA,5)))
}

if(is.na(res_owl_rbf_aug$stage1$beta0)){
  return(list(s=s,Tree=rep(NA,5),Alearn=rep(NA,5),Qlearn=rep(NA,5),
              owl_lin=rep(NA,5),owl_lin_aug=rep(NA,5),owl_rbf=rep(NA,5)))
}

mean_opt_owl_rbf=mean.opt.owl(res_owl_rbf_aug,'rbf',B)
 ###################################ASSESS######################################
 #################Assess the performance of each method on the testing set
 opt1=as.numeric((data_tes$L11>30)|(data_tes$L12>12))
 opt2=as.numeric((data_tes$L21>25)|(data_tes$L22>10))
 opt3=as.numeric((data_tes$L31+data_tes$L32)>35)

 ###assess the performance of causal tree
 contra1_tree=predict(tree1,newdata = data_tes)
 contra2_tree=predict(tree2,newdata = data_tes)
 contra3_tree=predict(tree3,newdata = data_tes)
 ###decision accuracy of causal tree
 accu1=mean(opt1==as.numeric(contra1_tree>0))
 accu2=mean(opt2==as.numeric(contra2_tree>0))
 accu3=mean(opt3==as.numeric(contra3_tree>0))

 accu=mean((opt1==as.numeric(contra1_tree>0))&(opt2==as.numeric(contra2_tree>0))
          &(opt3==as.numeric(contra3_tree>0)))

 ###assess the performance of A-learning
 blip_mat1=cbind(numeric(n_tes)+1,data_tes$W,data_tes$L11,data_tes$L12)
```

```
 blip_mat2=cbind(numeric(n_tes)+1,blip_mat1[,-1],data_tes$A1,data_tes$L21,data_tes$L22)
 blip_mat3=cbind(numeric(n_tes)+1,blip_mat2[,-1],data_tes$A2,data_tes$L31,data_tes$L32)

 contra1_dr=blip_mat1%*%psi_dr[[1]]
 contra2_dr=blip_mat2%*%psi_dr[[2]]
 contra3_dr=blip_mat3%*%psi_dr[[3]]
 ###decision accuracy of A-learning
 accu1_dr=mean(opt1==as.numeric(contra1_dr>0))
 accu2_dr=mean(opt2==as.numeric(contra2_dr>0))
 accu3_dr=mean(opt3==as.numeric(contra3_dr>0))

 accu_dr=mean((opt1==as.numeric(contra1_dr>0))&(opt2==as.numeric(contra2_dr>0))&
                 (opt3==as.numeric(contra3_dr>0)))

 ###assess the performance of Q-learning
 contra1_q=blip_mat1%*%psi_q[[1]]
 contra2_q=blip_mat2%*%psi_q[[2]]
 contra3_q=blip_mat3%*%psi_q[[3]]
 ###decision accuracy of Q-learning
 accu1_q=mean(opt1==as.numeric(contra1_q>0))
 accu2_q=mean(opt2==as.numeric(contra2_q>0))
 accu3_q=mean(opt3==as.numeric(contra3_q>0))

 accu_q=mean((opt1==as.numeric(contra1_q>0))&(opt2==as.numeric(contra2_q>0))
             &(opt3==as.numeric(contra3_q>0)))

#####assess the performance of OWL methods on the test set ############
A3_tes=2*data_tes$A3-1
A2_tes=2*data_tes$A2-1
A1_tes=2*data_tes$A1-1

H1_test=scale(cbind(data_tes$W,data_tes$L11,data_tes$L12))
H2_test=cbind(H1_test,A1_tes,scale(cbind(data_tes$L21,data_tes$L22)))
H3_test=cbind(H2_test,A2_tes,scale(cbind(data_tes$L31,data_tes$L32)))

########assess O1
tes_owl_lin=predict(res_owl_lin,H=list(H1_test,H2_test,H3_test),K=3)
accu3_lin=mean(tes_owl_lin$treatment[[3]]==(2*opt3-1))
accu2_lin=mean(tes_owl_lin$treatment[[2]]==(2*opt2-1))
accu1_lin=mean(tes_owl_lin$treatment[[1]]==(2*opt1-1))
accu_lin=mean((tes_owl_lin$treatment[[3]]==(2*opt3-1))&
            (tes_owl_lin$treatment[[2]]==(2*opt2-1))&
            (tes_owl_lin$treatment[[1]]==(2*opt1-1)))

########assess O2
tes_owl_lin_aug=predict(res_owl_lin_aug,H=list(H1_test,H2_test,H3_test),K=3)
accu3_lin_aug=mean(tes_owl_lin_aug$treatment[[3]]==(2*opt3-1))
accu2_lin_aug=mean(tes_owl_lin_aug$treatment[[2]]==(2*opt2-1))
accu1_lin_aug=mean(tes_owl_lin_aug$treatment[[1]]==(2*opt1-1))
accu_lin_aug=mean((tes_owl_lin_aug$treatment[[3]]==(2*opt3-1))&
                (tes_owl_lin_aug$treatment[[2]]==(2*opt2-1))&
                (tes_owl_lin_aug$treatment[[1]]==(2*opt1-1)))
```

```
  ########assess O3
  tes_owl_rbf_aug=predict(res_owl_rbf_aug,H=list(H1_test,H2_test,H3_test),K=3)
  accu3_rbf=mean(tes_owl_rbf_aug$treatment[[3]]==(2*opt3-1))
  accu2_rbf=mean(tes_owl_rbf_aug$treatment[[2]]==(2*opt2-1))
  accu1_rbf=mean(tes_owl_rbf_aug$treatment[[1]]==(2*opt1-1))
  accu_rbf=mean((tes_owl_rbf_aug$treatment[[3]]==(2*opt3-1))&
                (tes_owl_rbf_aug$treatment[[2]]==(2*opt2-1))&
                (tes_owl_rbf_aug$treatment[[1]]==(2*opt1-1)))

  return(list(s=s,
              Tree=c(accu1,accu2,accu3,accu,mean_opt_tree),
              Alearn=c(accu1_dr,accu2_dr,accu3_dr,accu_dr,mean_opt_dr),
              Qlearn=c(accu1_q,accu2_q,accu3_q,accu_q,mean_opt_q),
              owl_lin=c(accu1_lin,accu2_lin,accu3_lin,accu_lin, mean_opt_owl_lin),
              owl_lin_aug=c(accu1_lin_aug,accu2_lin_aug,accu3_lin_aug,accu_lin_aug,
                            mean_opt_owl_lin_aug),
              owl_rbf=c(accu1_rbf,accu2_rbf,accu3_rbf,accu_rbf, mean_opt_owl_rbf)))
}

sim.case2(1,n_tra=100,n_tes=1000,B=10000)
```

```
## [1] 1
```

```
## $s
## [1] 1
##
## $Tree
## [1]  0.68700  0.69100  0.71300  0.25600 93.03665
##
## $Alearn
## [1]  0.72900  0.82700  0.76700  0.46500 94.58258
##
## $Qlearn
## [1]  0.78000  0.82000  0.64900  0.42100 93.76578
##
## $owl_lin
## [1]  0.68700  0.79500  0.83400  0.42700 94.89128
##
## $owl_lin_aug
## [1]  0.5040  0.7790  0.5800  0.2340 91.4238
##
## $owl_rbf
## [1]  0.67800  0.44700  0.64800  0.27300 90.50096
```

References

Wallace, Michael P, and Erica EM Moodie. 2015. "Doubly-Robust Dynamic Treatment Regimen Estimation via Weighted Least Squares." *Biometrics* 71 (3): 636–44.
