# Supplementary material for: A Tutorial on Optimal Dynamic Treatment Regimes
Source: Stat Med. 2026 Feb 4;45(3-5):e70395. doi: 10.1002/sim.70395 (PMC12872042; doi:10.1002/sim.70395)
Supplement: Supplementary file 1 — Data S1: Additional supporting information may be found in the online version in the Supporting Information section at the end of this article. [file SIM-45-0-s001.zip › supplement_Wang&Tom.pdf]

Supplement to 'A tutorial on optimal dynamic treatment regimes'

Chunyu Wang and Brian DM Tom

S1: Sandwich variance estimator

We now illustrate the calculation of the sandwich variance estimator of the asymptotic variance of A-learning methods. Take A3 as an example.

S1.1 Stage 2

The estimating equation (EE) in stage 2, as shown in Section 4.1 in the tutorial, is

$$\sum_{i=1}^{n} M_i^{\psi_2}(\psi_2, \xi_2, \hat{\alpha}_2) = \sum_{i=1}^{n} R_{2i}\left[Y_i - A_{2i}R_{2i}^{\top}\psi_2 - D_{2i}^{\top}\xi_2\right]\left[A_{2i} - \mathbb{E}(A_{2i} \mid H_{2i}; \hat{\alpha}_2)\right] = 0, \tag{S.1}$$

and

$$\sum_{i=1}^{n} M_i^{\xi_2}(\psi_2, \xi_2) = \sum_{i=1}^{n} D_{2i}\left[Y_i - A_{2i}R_{2i}^{\top}\psi_2 - D_{2i}^{\top}\xi_2\right] = 0, \tag{S.2}$$

where $\mathbb{E}(A_{2i} \mid H_{2i}; \hat{\alpha}_2) = \text{expit}(R_{2i}^{\top}\hat{\alpha}_2)$ and $\hat{\alpha}_2$ is obtained from

$$\sum_{i=1}^{n} M_i^{\alpha_2}(\alpha_2) = \sum_{i=1}^{n} R_{2i}\left[A_{2i} - \mathbb{E}(A_{2i} \mid H_{2i}; \alpha_2)\right] = 0. \tag{S.3}$$

Let $\hat{\xi}_2(\psi_2)$ be the solution to equation (S.2) and $\xi_2^*(\psi_2)$ be the limiting form of $\hat{\xi}_2(\psi_2)$. To get the sandwich variance estimator of the asymptotic variance of $\hat{\psi}_2$, we need to apply the Taylor expansion to $M_i^{\psi_2}(\hat{\psi}_2, \hat{\xi}_2, \hat{\alpha}_2)$ around the limiting value of $(\hat{\psi}_2, \hat{\xi}_2(\hat{\psi}_2), \hat{\alpha}_2)$, say $\theta_2^* = (\psi_2^*, \xi_2^*(\psi_2^*), \alpha_2^*)$. Specifically,

$$\begin{aligned}
0 =& n^{-1/2}\sum_{i=1}^{n} M_i^{\psi_2}(\hat{\psi}_2, \hat{\xi}_2, \hat{\alpha}_2) \\
=& n^{-1/2}\sum_{i=1}^{n}\Big[M_i^{\psi_2}(\theta_2^*) + \Big(\frac{\partial M_i^{\psi_2}}{\partial \psi_2}\Big|_{\theta_2^*} + \frac{\partial M_i^{\psi_2}}{\partial \xi_2}\Big|_{\theta_2^*}\frac{\partial \hat{\xi}_2}{\partial \psi_2}\Big|_{\theta_2^*}\Big)(\hat{\psi}_2 - \psi_2^*) \\
&+ \frac{\partial M_i^{\psi_2}}{\partial \alpha_2}\Big|_{\theta_2^*}(\hat{\alpha}_2 - \alpha_2^*) + \frac{\partial M_i^{\psi_2}}{\partial \xi_2}\Big|_{\theta_2^*}\big(\hat{\xi}_2(\psi_2^*) - \xi_2^*(\psi_2^*)\big)\Big] + o_p(1),
\end{aligned} \tag{S.4}$$

where

$$\sqrt{n}(\hat{\alpha}_2 - \alpha_2^*) = -\left(\mathbb{E}\left[\frac{dM_i^{\alpha_2}}{d\alpha_2}\Big|_{\alpha_2^*}\right]\right)^{-1} n^{-1/2}\sum_{i=1}^{n} M_i^{\alpha_2}(\alpha_2^*) + o_p(1), \tag{S.5}$$

as implied by equation (S.3);

$$\sqrt{n}\left(\hat{\xi}_2(\psi_2^*) - \xi_2^*(\psi_2^*)\right) = -\left(\mathbb{E}\left[\frac{\partial M_i^{\xi_2}}{\partial \xi_2}\Big|_{(\psi_2^*, \xi_2^*(\psi_2^*))}\right]\right)^{-1} n^{-1/2}\sum_{i=1}^{n} M_i^{\xi_2}(\psi_2^*, \xi_2^*(\psi_2^*)) + o_p(1), \tag{S.6}$$

as implied by equation (S.2);

$$\frac{1}{n}\sum_{i=1}^{n}\frac{\partial M_i^{\psi_2}}{\partial \psi_2}\Big|_{\theta_2^*} \xrightarrow{P} \mathbb{E}\left[\frac{\partial M_i^{\psi_2}}{\partial \psi_2}\Big|_{\theta_2^*}\right], \tag{S.7}$$

$$\frac{1}{n}\sum_{i=1}^{n}\frac{\partial M_i^{\psi_2}}{\partial \xi_2}\Big|_{\theta_2^*} \xrightarrow{P} \mathbb{E}\left[\frac{\partial M_i^{\psi_2}}{\partial \xi_2}\Big|_{\theta_2^*}\right], \tag{S.8}$$

$$\frac{1}{n}\sum_{i=1}^{n}\frac{\partial M_i^{\psi_2}}{\partial \alpha_2}\Big|_{\theta_2^*} \xrightarrow{P} \mathbb{E}\left[\frac{\partial M_i^{\psi_2}}{\partial \alpha_2}\Big|_{\theta_2^*}\right], \tag{S.9}$$

due to the law of large numbers; and

$$\frac{\partial \hat{\xi}_2}{\partial \psi_2}\Big|_{\theta_2^*} \xrightarrow{P} \frac{\partial \xi_2^*}{\partial \psi_2}\Big|_{\theta_2^*}. \tag{S.10}$$

Substituting (S.5)-(S.10) into (S.4), we get

$$\begin{aligned}\sqrt{n}(\hat{\psi}_2 - \psi_2^*) = &\left(\mathbb{E}\left[\frac{\partial M_i^{\psi_2}}{\partial \psi_2}\Big|_{\theta_2^*}\right] + \mathbb{E}\left[\frac{\partial M_i^{\psi_2}}{\partial \xi_2}\Big|_{\theta_2^*}\right]\frac{\partial \xi_2^*}{\partial \psi_2}\Big|_{\theta_2^*}\right)^{-1} \times \\ n^{-1/2}\sum_{i=1}^{n}&\left\{M_i^{\psi_2}(\theta_2^*) - \mathbb{E}\left[\frac{\partial M_i^{\psi_2}}{\partial \alpha_2}\Big|_{\theta_2^*}\right]\left(\mathbb{E}\left[\frac{dM_i^{\alpha_2}}{d\alpha_2}\Big|_{\alpha_2^*}\right]\right)^{-1} M_i^{\alpha_2}(\alpha_2^*)\right. \\ &\left. - \mathbb{E}\left[\frac{\partial M_i^{\psi_2}}{\partial \xi_2}\Big|_{\theta_2^*}\right]\left(\mathbb{E}\left[\frac{\partial M_i^{\xi_2}}{\partial \xi_2}\Big|_{(\psi_2^*, \xi_2^*(\psi_2^*))}\right]\right)^{-1} M_i^{\xi_2}(\psi_2^*, \xi_2^*(\psi_2^*))\right\} + o_p(1).\end{aligned} \tag{S.11}$$

Therefore, the sandwich variance estimator of the asymptotic variance of $\hat{\psi}_2$ is

$$
\left(\mathbb{E}\left[\frac{\partial M_i^{\psi_2}}{\partial \psi_2}\Big|_{\theta_2^*}\right] + \mathbb{E}\left[\frac{\partial M_i^{\psi_2}}{\partial \xi_2}\Big|_{\theta_2^*}\right]\frac{\partial \xi_2^*}{\partial \psi_2}\Big|_{\theta_2^*}\right)^{-1} \times
$$

$$
\begin{aligned}
\mathrm{Var}\Bigg\{ & M_i^{\psi_2}(\theta_2^*) - \mathbb{E}\left[\frac{\partial M_i^{\psi_2}}{\partial \alpha_2}\Big|_{\theta_2^*}\right]\left(\mathbb{E}\left[\frac{dM_i^{\alpha_2}}{d\alpha_2}\Big|_{\alpha_2^*}\right]\right)^{-1} M_i^{\alpha_2}(\alpha_2^*) \\
& - \mathbb{E}\left[\frac{\partial M_i^{\psi_2}}{\partial \xi_2}\Big|_{\theta_2^*}\right]\left(\mathbb{E}\left[\frac{\partial M_i^{\xi_2}}{\partial \xi_2}\Big|_{(\psi_2^*, \xi_2^*(\psi_2^*))}\right]\right)^{-1} M_i^{\xi_2}(\psi_2^*, \xi_2^*(\psi_2^*))\Bigg\} \times
\end{aligned}
$$

$$
\left(\mathbb{E}\left[\frac{\partial M_i^{\psi_2}}{\partial \psi_2}\Big|_{\theta_2^*}\right] + \mathbb{E}\left[\frac{\partial M_i^{\psi_2}}{\partial \xi_2}\Big|_{\theta_2^*}\right]\frac{\partial \xi_2^*}{\partial \psi_2}\Big|_{\theta_2^*}\right)^{-1}.
$$

S1.2 Stage 1

The estimating equation in stage 1 is

$$
\begin{aligned}
\sum_{i=1}^n M_i^{\psi_1}(\psi_1, \hat{\psi}_2, \xi_1, \hat{\alpha}_1) = \sum_{i=1}^n R_{1i}\Big[Y_i + \mu_2(H_{2i}, A_{2i}; \hat{\psi}_2) - A_{1i}R_{1i}^\top\psi_1 - D_{1i}^\top\xi_1\Big] \\
\times \left[A_{1i} - \mathbb{E}(A_{1i} \mid H_{1i}; \hat{\alpha}_1)\right] = 0;
\end{aligned}
\tag{S.12}
$$

and

$$
\sum_{i=1}^n M_i^{\xi_1}(\psi_1, \hat{\psi}_2, \xi_1) = \sum_{i=1}^n D_{1i}\Big[Y_i + \mu_2(H_{2i}, A_{2i}; \hat{\psi}_2) - A_{1i}R_{1i}^\top\psi_1 - D_{1i}^\top\xi_1\Big] = 0, \tag{S.13}
$$

where $\mu_2(H_{2i}, A_{2i}; \hat{\psi}_2) = (I\{R_{2i}^\top\hat{\psi}_2 > 0\} - A_{2i})R_{2i}^\top\hat{\psi}_2$ and $\mathbb{E}(A_{1i} \mid H_{1i}; \hat{\alpha}_1) = \mathrm{expit}(R_{1i}^\top\hat{\alpha}_1)$; and $\hat{\alpha}_1$ is obtained from

$$
\sum_{i=1}^n M_i^{\alpha_1}(\alpha_1) = \sum_{i=1}^n R_{1i}\left[A_{1i} - \mathbb{E}(A_{1i} \mid H_{1i}; \alpha_1)\right] = 0. \tag{S.14}
$$

Let $\hat{\xi}_1(\psi_1, \hat{\psi}_2)$ be the solution to equation (S.13) and $\xi_1^*(\psi_1, \hat{\psi}_2)$ be the limiting form of $\hat{\xi}_1(\psi_1, \hat{\psi_2})$. Applying the Taylor expansion to $M_i^{\psi_1}$ around the limiting value of $(\hat{\psi}_1, \hat{\psi}_2, \hat{\xi}_1(\hat{\psi}_1, \hat{\psi}_2), \hat{\alpha}_1)$, say $\theta_1^* = (\psi_1^*, \psi_2^*, \xi_1^*(\psi_1^*, \psi_2^*), \alpha_1^*)$, yields

$$
\begin{aligned}
0 =& n^{-1/2}\sum_{i=1}^n M_i^{\psi_1}(\hat{\psi}_1, \hat{\psi}_2, \hat{\xi}_1, \hat{\alpha}_1) \\
=& n^{-1/2}\sum_{i=1}^n \Big[M_i^{\psi_1}(\theta_1^*) + \Big(\frac{\partial M_i^{\psi_1}}{\partial \psi_1}\Big|_{\theta_1^*} + \frac{\partial M_i^{\psi_1}}{\partial \xi_1}\Big|_{\theta_1^*}\frac{\partial \hat{\xi}_1}{\partial \psi_1}\Big|_{\theta_1^*}\Big)(\hat{\psi}_1 - \psi_1^*) \\
& + \frac{\partial M_i^{\psi_1}}{\partial \alpha_1}\Big|_{\theta_1^*}(\hat{\alpha}_1 - \alpha_1^*) + \frac{\partial M_i^{\psi_1}}{\partial \xi_1}\Big|_{\theta_1^*}\big(\hat{\xi}_1(\psi_1^*, \psi_2^*) - \xi_1^*(\psi_1^*, \psi_2^*)\big) \\
& + \Big(\frac{\partial M_i^{\psi_1}}{\partial \psi_2} + \frac{\partial M_i^{\psi_1}}{\partial \xi_1}\Big|_{\theta_1^*}\frac{\partial \hat{\xi}_1}{\partial \psi_2}\Big|_{\theta_1^*}\Big)(\hat{\psi}_2 - \psi_2^*)\Big] + o_p(1),
\end{aligned}
\tag{S.15}
$$

where $\sqrt{n}(\hat{\alpha}_1 - \alpha_1^*)$ and $\sqrt{n}\big(\hat{\xi}_1(\psi_1^*, \psi_2^*) - \xi_1^*(\psi_1^*, \psi_2^*)\big)$ can be derived in a similar way to that of (S.5) and (S.6), respectively; and $\sqrt{n}(\hat{\psi}_2 - \psi_2^*)$ has already been obtained in stage 2, as expressed in (S.11). Similar to the derivation of (S.11) from (S.4), we can derive the expression of $\sqrt{n}(\hat{\psi}_1 - \psi_1^*)$ from (S.15) and therefore get the sandwich variance estimator of the asymptotic variance of $\hat{\psi}_1$.

S2: Consistency of dWOLS

Consider a single stage case where the conditional expectation of Y is

$$\mathbb{E}[Y|L, A] = A(\psi_0^* + \psi_1^* L) + m(L).$$

Let $R_i = (1, L_i)^\top$. Consider a working model for $E[Y - A(\psi_0^* + \psi_1^* L)|L]$, for example, $\xi_0 + \xi_1 L + \xi_2 L^2$; and let $\tilde{R}_i = (1, L_i, L_i^2)^\top$. Solving the following equations

$$\begin{aligned} &\sum_{i=1}^{n} R_i \left[Y_i - A_i R_i^\top \psi - \tilde{R}_i \xi_i\right] \left[A_i - \hat{\mathbb{E}}\left(A_i|L_i\right)\right] = 0, \\ &\sum_{i=1}^{n} \tilde{R}_i \left[Y_i - A_i R_i^\top \psi - \tilde{R}_i \xi_i\right] = 0; \end{aligned} \tag{S.16}$$

gives a consistent estimate for $\psi^* = (\psi_0^*, \psi_1^*)$ due to the fact $\mathbb{E}[Y - A(\psi_0^* + \psi_1^* L)|L, A] = \mathbb{E}[Y - A(\psi_0^* + \psi_1^* L)|L]$ whatever $m(\cdot)$ is. Let $\{(Y_i^w, L_i^w, A_i^w)\}_{i=1}^n$ denote the weighted data such that $A^w \perp L^w$. See Wallace and Moodie (2015) for choices of the weight function. Applying the equations in (S.16) to the weighted data, we get

$$\sum_{i=1}^{n} R_i^w \left[Y_i^w - A_i^w R_i^{w\top} \psi - \tilde{R}_i^w \xi_i\right] \left[A_i^w - \hat{\mathbb{E}}\left(A_i^w\right)\right] = 0, \tag{S.17a}$$

$$\sum_{i=1}^{n} \tilde{R}_i^w \left[Y_i^w - A_i^w R_i^{w\top} \psi - \tilde{R}_i^w \xi_i\right] = 0. \tag{S.17b}$$

Note that $\hat{\mathbb{E}}\left(A_i^w\right)$ is a constant estimated from the weighted data and that all elements of R_i^w are included in $\tilde{R}_i^w$. Substituting (S.17b) into (S.17a) gives

$$\begin{aligned} &\sum_{i=1}^{n} R_i^w \left[Y_i^w - A_i^w R_i^{w\top} \psi - \tilde{R}_i^w \xi_i\right] A_i^w = 0, \\ &\sum_{i=1}^{n} \tilde{R}_i^w \left[Y_i^w - A_i^w R_i^{w\top} \psi - \tilde{R}_i^w \xi_i\right] = 0; \end{aligned}$$

which is exactly regressing Y^w on $A^w R^w$ and $\tilde{R}^w$. Therefore, the weighted OLS regression of Y on AR and $\tilde{R}$ yields consistent estimates of ψ^*.

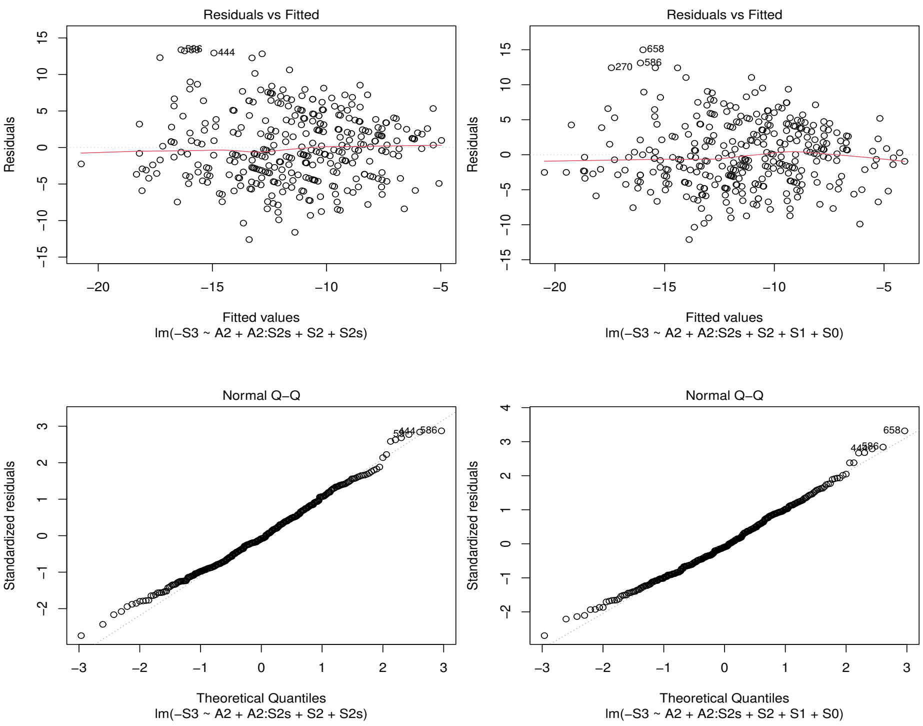

Figure S1: Diagnostics for the regression models specified for the Q-function in stage 2. Left: the treatment-free term is specified as $1 + S_2 + \Delta S_2$. Right: the treatment-free term is specified as $1 + S_2 + S_1 + S_0$. 'S2s' in the function lm denotes the slope of S_2, i.e., ΔS_2.

S3: additional results for STAR*D analysis

References

Wallace, M. P. and Moodie, E. E. (2015). Doubly-robust dynamic treatment regimen estimation via weighted least squares. *Biometrics*, 71(3):636–644.

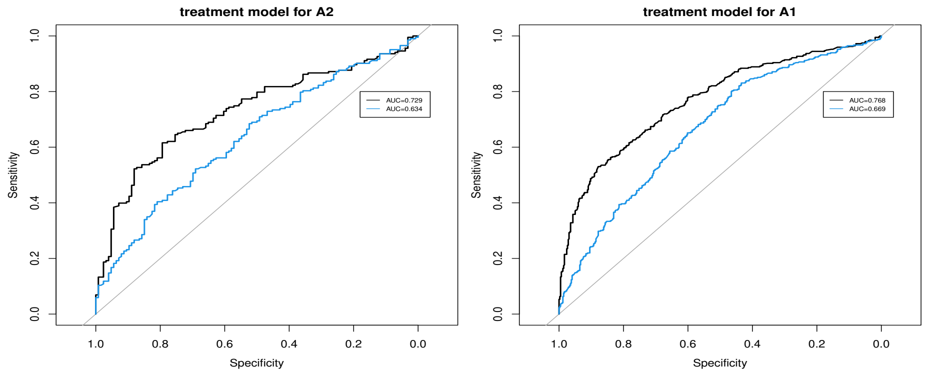

Figure S2: Diagnostics for the logistic regression models specified for the propensity scores in stage 2 (left) and stage 1 (right). The black ROC curves correspond to the model $P(A_2 = 1|\bar{L}_2, I_{\text{sim}} = 0, A_1; \alpha_2) = \text{expit}(\alpha_{20} + \alpha_{21}S_2 + \alpha_{22}T_1 + \alpha_{23}A_1)$ in the left and the model $P(A_1 = 1|\bar{L}_1; \alpha_1) = \text{expit}(\alpha_{10} + \alpha_{11}S_1 + \alpha_{12}T_0)$ in the right.The blue ROC curves correspond to the model $P(A_2 = 1|\bar{L}_2, I_{\text{sim}} = 0, A_1; \alpha_2) = \text{expit}(\alpha_{20} + \alpha_{21}S_2 + \alpha_{22}\Delta S_2 + \alpha_{23}A_1)$ in the left and the model $P(A_1 = 1|\bar{L}_1; \alpha_1) = \text{expit}(\alpha_{10} + \alpha_{11}S_1 + \alpha_{12}\Delta S_1)$ in the right.

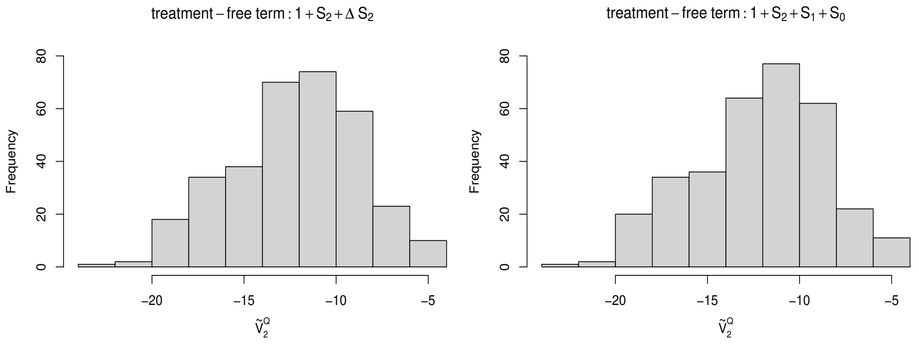

Figure S3: Histograms of the values of $\tilde{V}_2^Q$ under different treatment-free terms.

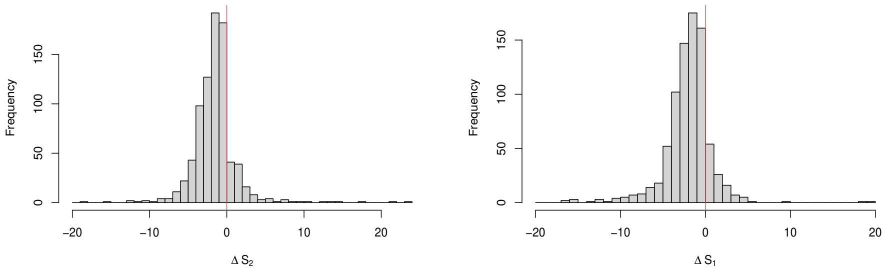

Figure S4: Left: the histogram of the slope of QIDS-C_{16} over level 2, i.e., ΔS_2. Right: the histogram of the slope of QIDS-C_{16} over level 1, i.e., ΔS_1.

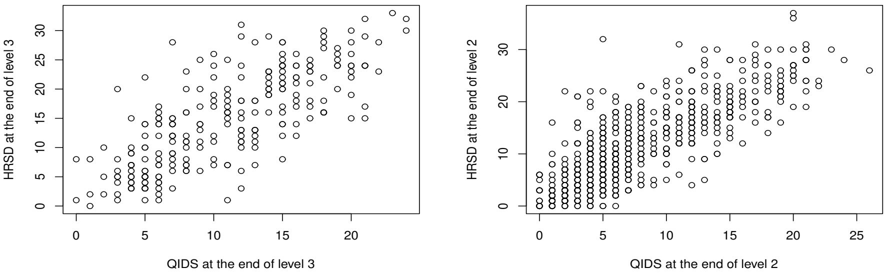

Figure S5: Correlation between QIDS-C_{16} and $HRSD_{17}$.
